# Maternal pesticide exposure and risk of birth defects: a population-based cross-sectional study in China

**DOI:** 10.3389/fpubh.2024.1489365

**Published:** 2024-12-06

**Authors:** Fangfang Liu, Xiayang Li, Jie Chen, Yishuai Huang, Shaonong Dang

**Affiliations:** ^1^Department of Stomatology, Xi’an Central Hospital, Xi’an, Shaanxi, China; ^2^Department of Epidemiology and Health Statistics, School of Public Health, Xi’an Jiaotong University Health Science Center, Xi’an, Shaanxi, China

**Keywords:** pesticides, birth defects, perinatal pregnancy, cardiovascular system defects, crosssectional survey

## Abstract

**Objective:**

This study aimed to examine the association between maternal pesticide exposure during the periconceptional period and birth defects in their offspring.

**Methods:**

A survey was conducted among 29,204 women with infants born between 2010 and 2013 in Shaanxi Province, Northwest China. All cases of birth defects were diagnosed using the International Classification of Diseases, Tenth Revision (ICD-10). Given the multistage sampling design, the generalized estimating equation (GEE) binomial regression models with log link and exchangeable correlation structures were used to analyze the association between maternal pesticide exposures and birth defects.

**Results:**

Among the 29,204 subjects, 562 mothers had children with birth defects, resulting in an incidence rate of 192.44 per 10,000 live births. The incidence of birth defects was higher in the pesticide-exposed group compared to the control group (737.46/10,000 vs. 186.04/10,000). After adjusting for baseline demographic characteristics, fertility status, nutritional factors, and environmental factors in the GEE model, the results indicated that the risk of birth defects and cardiovascular system defects in mothers exposed to pesticides during the periconceptional period was 2.39 times (95% CI: 1.84–3.10) and 3.14 times (95% CI: 1.73–5.71) higher, respectively, compared to the control group.

**Conclusion:**

This study demonstrated that maternal exposure to pesticides during the periconceptional period was associated with an increased risk of birth defects, particularly cardiovascular system defects in offspring. Consequently, it would be beneficial to avoid pesticide exposure from three months before pregnancy through the first trimester to lower birth defects in infants.

## 1 Introduction

Congenital abnormalities, commonly known as birth defects, include a wide range of anatomical and functional irregularities that develop during embryonic or fetal growth. The International Classification of Diseases, Tenth Revision (ICD-10), identifies approximately 10 systems in which birth defects can occur. Over 20 significant birth defects are frequently encountered in clinical practice, including congenital heart disease, neural tube defects, limb shortening, cleft lip and palate, and trisomy 21 syndrome. In China, the prevalence of birth defects was 123 per 10,000 perinatal births during 2000–2021 (1). These defects contribute significantly to early abortion, stillbirth, perinatal death, infant mortality, and congenital disabilities (2).

A growing body of evidence suggests that the risk of birth defects is influenced by a complex interaction of genetic and environmental factors. The widespread use of pesticides, especially in agriculture, has been shown through experimental research to produce endocrine disruptors, neurodevelopmental toxicants, immunotoxicants, and carcinogens (3, 4). Numerous studies, both domestic and international, indicate that prenatal exposure to pesticides may increase the risk of adverse birth outcomes, including miscarriage, premature delivery, stillbirth, and birth defects (5–9). These exposures may also impact the long-term cognitive and physical development of offspring (10–13). A case–control study in Northern Netherlands identified maternal occupational exposure to pesticides and dust as potential risk factors for oral clefts in offspring (14). Additionally, exposure to personal, household, and agricultural pesticides during pregnancy may raise the risk of holoprosencephaly (15). An animal study provides new insights into the harmful effects of pesticide and environmental chemical exposure on craniofacial skeletal development in zebrafish embryos (16).

However, some studies have produced conflicting results regarding the correlation between prenatal pesticide exposure and birth defects. For instance, a case–control study in California found no evidence of a correlation between maternal residential exposure to agricultural pesticides and specific congenital heart defects in children (17). Moreover, most pesticides analyzed did not show a statistically significant relationship with an increased risk of hypospadias in male infants (18). These conflicts might result from differences in pesticide exposure assessment methods and study population heterogeneity (19). Different agricultural practices, pesticide types, genetic backgrounds in various regions, as well as small sample sizes in some studies and possible protective genetic factors in certain areas with low pesticide usage, can make the exposure birth defect association less obvious and reduce statistical power (20, 21).

The period extending from 3 months prior to conception through the first trimester of pregnancy is critical for both conception and fetal development. Notably, during the first trimester, pregnant women are particularly susceptible to the effects of pesticides due to physiological changes and the immature state of the placenta (22). In many agricultural regions of low- and middle-income countries, pesticide use coincides with the periconceptional stage, increasing exposure risk (23). Pesticide exposure in the early stage of life can also lead to long term issues like developmental delays, autism spectrum disorder and learning disabilities (24). Given the current understanding of the link between maternal pesticide exposure during early pregnancy and birth defects in offspring, further research is needed.

Therefore, this study used data from a large population-based survey of birth defects conducted in Shaanxi Province, Northwest China, to explore the potential connection between maternal pesticide exposure during the periconceptional period and the occurrence of birth defects in offspring. The findings of this study could help inform strategies for the prevention and management of birth defects.

## 2 Methods

### 2.1 Study design and participants

This study used data from a population-based cross-sectional survey on birth defects conducted in Shaanxi Province, Northwest China, between July and November 2013. Mothers with documented pregnancy outcomes and their infants born between 2010 and 2013 were selected using a stratified multistage sampling approach. The detailed sampling methodology has been previously published (25). The study was approved by the Human Research Ethics Committee of Xi’an Jiaotong University (approval number: 2012008). Written informed consent was obtained from all participants. After excluding 823 subjects due to unclear pregnancy outcomes or incomplete questionnaires, the final analysis included 29,204 women.

### 2.2 Definition and diagnosis of birth defects

Birth defects were classified according to ICD-10 codes, covering malformations of various body systems, including defects of the nervous system; eyes, ears, face, and neck; circulatory system; respiratory system; cleft lip and palate; digestive system; reproductive organs; urinary system; musculoskeletal system; other congenital malformations; and chromosomal abnormalities not classified elsewhere.

An expert team, including senior medical technicians from the departments of congenital heart surgery, ultrasound, obstetrics, and gynecology at the First Affiliated Hospital of Xi’an Jiaotong University, was assembled for the diagnosis of birth defects. The diagnosis of congenital anomalies was determined by this team using the ICD-10. For pediatric patients with external anomalies, medical records were reviewed, and malformations were documented with photographic evidence to aid in diagnosis. For congenital internal malformations, including cardiovascular anomalies, medical records were reviewed, and supplementary ultrasound examinations were conducted at the First Affiliated Hospital of Xi’an Jiaotong University to establish a definitive diagnosis.

### 2.3 Definition of pesticide exposure and the periconceptional period

Pesticides included various chemical classes such as pyrethroids, carbamates, organophosphorus compounds, organofluorides, organochlorines, avermectin, carbendazim, dafenazine, sofril, rodenticides, and herbicides, among others. The periconceptional period was defined as the span from 3 months before pregnancy to the first 3 months of pregnancy.

### 2.4 Definitions of covariates

Previous research (26–28) has identified several factors correlated with birth defects, including maternal socio-demographic characteristics, fertility status, nutritional factors, and environmental factors. Specifically, these variables included maternal age (<30 and ≥ 30 years), education level (college degree and above, high school or technical secondary school, junior high school, primary school and below), ethnicity (Han, other), residence (rural, urban), household wealth index (poor, moderate, rich), first pregnancy (yes, no), history of abortions (yes, no), type of pregnancy (singleton, multiple), family history of birth defects (yes, no), folic acid supplementation (yes, no), infections (yes, no), tobacco exposure (yes, no), occupational risk exposure (yes, no), and industrial exposure (yes, no).

The folic acid supplementation regimen was defined as the consistent intake of at least 400 μg of folic acid per day during the periconceptional period, sustained for more than three consecutive months. Infection was characterized by febrile episodes (temperature exceeding 38°C) and symptoms related with influenza or the common cold, including influenza-like symptoms and seasonal influenza during pregnancy. Tobacco exposure during pregnancy was categorized into two groups: active smoking, defined as the consumption of at least one cigarette per week for 3 consecutive months, and passive smoking, defined as inhaling secondhand smoke for a minimum of 15 min per day over one consecutive month. Occupational exposure was defined by the presence of biological, physical, chemical, or psychosocial risk factors in the workplace. Industrial exposure was defined as residing within a one-kilometer radius of mines, cement factories, power plants, fertilizer factories, or major traffic thoroughfares during pregnancy.

### 2.5 Data collection

A standardized questionnaire was developed by the Department of Epidemiology and Health Statistics at the School of Public Health, Xi’an Jiaotong University Health Science Center. This questionnaire gathered information on maternal socio-demographic characteristics, fertility status, nutritional factors, and environmental factors during pregnancy, and child birth outcomes as reported by the mothers. Trained field staff conducted face-to-face interviews to administer the questionnaire. Additionally, diagnostic information regarding birth outcomes, including the timing of diagnosis and types of birth defects, was collected from local hospitals. Data on perinatal pesticide exposure were self-reported by mothers, detailing contact with pesticides via skin or respiratory routes during this period.

### 2.6 Statistical analysis

Categorical variables were summarized as frequencies and percentages, and intergroup comparisons were performed using the chi-squared test, corrected chi-squared test, or Fisher’s exact test, as appropriate. Continuous variables were reported as mean and standard deviation, with group comparisons conducted using the *t*-test for normally distributed variables or the Wilcoxon rank-sum test for non-normally distributed variables. The generalized estimating equation (GEE) extends the generalized linear model, specifically addressing the analysis of non-independent data. The GEE is adept at fitting suitable statistical models to dependent variables that conform to a variety of distributions, such as normal, binomial, and Poisson. This statistical method effectively mitigates the issue of correlation among dependent variables in longitudinal datasets, thereby enhancing the reliability of the estimates obtained. Given the multistage sampling design, the GEE was used to analyze the association between maternal pesticide exposures during the periconceptional period and the incidence of birth defects, aiming to mitigate intra-group correlations. Additionally, to explore the potential heterogeneity in the effects of pesticide exposures on birth defects, effects were estimated within subgroups defined by various maternal characteristics.

The data entry process was conducted in duplicate with error checking using EpiData 3.1 software. Statistical analysis was performed using SAS 9.4 software (Statistics Analysis System, Inc., Cary, North Carolina, United States), with statistical significance set at a two-tailed *p*-value of less than 0.05.

## 3 Results

### 3.1 Participants’ characteristics

Table 1 and Supplementary Table S1 presents the baseline characteristics of the 29,204 pregnant women included in the study. The mean age of the participants was 27.09 ± 4.76 years. Most participants had a junior high school education level (49.60%), were of Han ethnicity (99.36%), and resided in rural areas (67.24%). Among the participants, 339 women were in the pesticide exposure group, while 28,865 were in the non-exposure group. Compared to the non-exposure group, women in the pesticide exposure group were more likely to be older, live in rural areas, have a history of abortion and infection, be exposed to tobacco, and encounter occupational and environmental risk factors. They also tended to have lower education levels and household wealth index and were less likely to take folic acid supplements. No significant differences were observed between the two groups in terms of maternal ethnicity, type of pregnancy and family history of birth defects.

**Table 1:** Baseline characteristics of participants.

Variables	Total	Pesticide exposure group	Non-pesticide exposure group	t/χ^2^/Z	*p* value
Maternal age, Mean ± SD	27.09 ± 4.76	30.05 ± 5.63	27.05 ± 4.74	9.907	<0.001^a^
Maternal age, *n* (%)					
<30 years	22,309 (76.39)	172 (50.74)	22,137 (76.69)	125.144	<0.001
≥30 years	6,895 (23.61)	167 (49.26)	6,728 (23.31)		
Maternal education level, *n* (%)					
Junior college and undergraduate or above	5,326 (18.24)	15 (4.42)	5,311 (18.40)	98.615	<0.001
High school	5,835 (19.98)	35 (10.32)	5,800 (20.09)		
Middle school	14,485 (49.60)	209 (61.65)	14,276 (49.46)		
Primary school or below	3,558 (12.18)	80 (23.60)	3,478 (12.05)		
Residence, *n* (%)					
Rural	19,637 (67.24)	314 (92.63)	19,323 (66.94)	100.333	<0.001
Urban	9,567 (32.76)	25 (7.37)	9,542 (33.06)		
Household wealth index, *n* (%)					
Poor	9,743 (33.36)	159 (46.90)	9,584 (33.20)	34.489	<0.001
Moderate	9,773 (33.46)	109 (32.15)	9,664 (33.48)		
Rich	9,688 (33.17)	71 (20.94)	9,617 (33.32)		
First pregnancy, *n* (%)					
Yes	14,926 (51.11)	68 (20.06)	14,858 (51.47)	132.337	<0.001
No	14,278 (48.89)	271 (79.94)	14,007 (48.53)		
History of abortions, *n* (%)					
Yes	4,057 (13.89)	91 (26.84)	3,966 (13.74)	48.097	<0.001
No	25,147 (86.11)	248 (73.16)	24,899 (86.26)		
Maternal folic acid supplementation, *n* (%)					
Yes	10,593 (36.27)	70 (20.65)	10,523 (36.46)	36.218	<0.001
No	18,611 (63.73)	269 (79.35)	18,342 (63.54)		
Maternal infections, *n* (%)					
Yes	3,821 (13.08)	77 (22.71)	3,744 (12.97)	27.970	<0.001
No	25,383 (86.92)	262 (77.29)	25,121 (87.03)		
Tobacco exposure, *n* (%)					
Yes	17,359 (59.44)	242 (71.39)	17,117 (59.30)	20.302	<0.001
No	11,845 (40.56)	97 (28.61)	11,748 (40.70)		
Occupational risk exposure, *n* (%)					
Yes	2,414 (8.27)	110 (32.45)	2,304 (7.98)	264.511	<0.001
No	26,790 (91.73)	229 (67.55)	26,561 (92.02)		
Industrial exposure, *n* (%)					
Yes	7,766 (26.59)	122 (35.99)	7,644 (26.48)	15.512	<0.001
No	21,438 (73.41)	217 (64.01)	21,221 (73.52)		

a Wilcoxon rank test.

### 3.2 Prevalence of birth defects

Table 2 shows the prevalence of birth defects among the 29,204 participants. A total of 562 women gave birth to children with birth defects, resulting in an overall incidence rate of 192.44 per 10,000 live births. The prevalence of birth defects (737.46/10,000 vs. 186.04/10,000), congenital malformations of the circulatory system (324.48/10,000 vs. 60.28/10,000), and other congenital malformations (147.49/10,000 vs. 29.79/10,000) was significantly higher in the pesticide-exposed group compared to the unexposed group.

**Table 2:** The prevalence of birth defects (1/10,000).

Types of birth defects	Total	Pesticide exposure group	Non-pesticide exposure group	χ^2^/Z	*p* value
Nervous system defect	20 (6.85)	1 (29.50)	19 (6.58)		0.208^a^
Eyes, ears, face and neck defect	70 (23.97)	2 (59.00)	68 (23.56)		0.195^a^
Circulatory system defect	185 (63.35)	11 (324.48)	174 (60.28)		<0.001^b^
Respiratory system defect	11 (3.77)	1 (29.50)	10 (3.46)		0.121^a^
Cleft lip and palate	34 (11.64)	2 (59.00)	32 (11.09)		0.059^a^
Digestive system defect	25 (8.56)	0 (0.00)	25 (8.66)		1.000^a^
Reproductive organs defect	17 (5.82)	1 (29.50)	16 (5.43)		0.180^a^
Urinary system defect	6 (2.05)	0 (0.00)	6 (2.08)		1.000^a^
Malformations and deformations of the musculoskeletal system	101 (34.58)	2 (59.00)	99 (27.38)		0.760^b^
Other congenital malformations	91 (31.16)	5 (147.49)	86 (29.79)		0.001^b^
Chromosomal abnormalities	2 (0.68)	0 (0.00)	2 (0.69)		1.000^a^
Birth defects (overall)	562 (192.44)	25 (737.46)	537 (186.04)	53.982	<0.001

a Fisher exact test.

b Continuity corrected χ^2^ test.

### 3.3 Pesticide exposure and birth defects

Due to the hierarchical structure of the data, with aggregation at the county level, a GEE model was used to account for the county (district) as a random effect in the multifactor analysis of pesticide exposure and birth defects (Table 3). In the unadjusted model (model 1), the risks of birth defects and cardiovascular system defects in the offspring of mothers exposed to pesticides were 4.20 times (95% CI: 3.16–5.59) and 5.53 times (95% CI: 3.14–9.73) higher, respectively, compared to the control group. After adjusting for baseline demographic characteristics (model 2), these risks decreased to 3.45 times (95% CI: 2.55–4.65) and 5.07 times (95% CI: 2.75–9.35) higher, respectively. Further adjustment for fertility status (model 3) reduced the risks to 3.23 times (95% CI: 2.40–4.33) and 4.70 times (95% CI: 2.52–8.77) higher, respectively. Finally, after adjusting for baseline demographic characteristics, fertility status, nutritional factors, and environmental factors (model 4), the risks of birth defects and cardiovascular system defects were 2.39 times (95% CI: 1.84–3.10) and 3.14 times (95% CI: 1.73–5.71) higher, respectively, compared to the control group.

**Table 3:** Association between pesticide exposure and birth defects.

	Model 1		Model 2^a^		Model 3^b^		Model 4^c^	
	Crude OR (95% CI)	*p* value	Adjusted OR (95% CI)	*p* value	Adjusted OR (95% CI)	*p* value	Adjusted OR (95% CI)	*p* value
Birth defects	4.20 (3.16–5.59)	<0.001	3.45 (2.55–4.65)	<0.001	3.23 (2.40–4.33)	<0.001	2.39 (1.84–3.10)	<0.001
Cardiovascular system defect	5.53 (3.14–9.73)	<0.001	5.07 (2.75–9.35)	<0.001	4.70 (2.52–8.77)	<0.001	3.14 (1.73–5.71)	<0.001

a Model 2 adjusted baseline demographic characteristics (maternal age, education level, ethnic, residence and household wealth index).

b Model 3 adjusted baseline demographic characteristics and fertility status (first pregnancy, history of abortions, type of pregnancy, and family history of birth defects).

c Model 4 adjusted baseline demographic characteristics, fertility status, nutritional factor (folic acid supplement), and environmental factors (infection, smoke or passive smoke, occupational exposure, and industrial exposure).

### 3.4 Sensitivity analyses

The relationship between pesticide exposure and birth defects was further examined across various demographic subgroups. Pesticide exposure was consistently associated with an increased risk of birth defects in all subgroups. This association was statistically significant for women under 30 years of age, women 30 years or older, those with a high school education or above, those with a middle school education or below, those living in rural areas, and those experiencing a non-first pregnancy. No significant interaction was observed between pesticide exposure and any covariates for birth defects (Supplementary Table S2).

## 4 Discussion

Drawing on a comprehensive population-based survey conducted in Shaanxi Province, Northwest China, this study investigated the relationship between pesticide exposure during the periconceptional period and the risk of birth defects. Using a GEE model, we adjusted for baseline demographic characteristics, fertility status, nutritional factors, and environmental factors. Our findings indicated that maternal pesticide exposure during the periconceptional period was significantly associated with an increased risk of birth defects, particularly congenital circulatory malformations, in offspring.

This study identified a correlation between maternal pesticide exposure during the periconceptional period and birth defects based on data from a cross-sectional survey conducted across 20 counties and 10 districts in Shaanxi Province, China. Our results were aligned with international findings in previous reports. For instance, a systematic review found that maternal exposure to pesticides during pregnancy is associated with a significantly increased risk of birth defects, with an odds ratio of 4.44 (95% CI: 2.61–7.57) compared to unexposed pregnant women (29). Evidence from a nested case–control study also suggested that pregnant women in southwest China were ubiquitously exposed to low-level pyrethroid pesticide (PYRs), and maternal household pesticides use was related to congenital anomalies (30). Similarly, a previous case control study conducted in a rural area of Northern China suggested that prenatal exposure to Organochlorine pesticides (OCPs) was associated with increased risk for neural tube defects (NTDs), the OCPs also had a joint effect on NTD as its risk increased almost linearly with concentrations of the 16 OCPs as a mixture (31). Another birth cohort study also revealed that exposure to pesticides in maternal residences increased the likelihood of congenital heart disease, reproductive system malformations, and skeletal muscle malformations in offspring (32). Unlike previous studies, this research specifically focused on pesticide exposure during the 3 months preceding pregnancy and the first trimester—a critical period for fetal development. The odds ratios of similar international studies were basically like those of this study, with case—control studies as the main research methods and maternal self-reporting often used for exposure assessment. Therefore, it is of great necessity to conduct large sample prospective studies and quantitatively measure the level of maternal pesticide exposure in the future to reveal the causal relationship between pesticides and birth defects.

In addition to the acute toxic effects of cholinergic toxicity, organophosphorus pesticides have been shown to cause genetic toxicity, including DNA damage, gene mutations, chromosome aberrations, and the induction of cell carcinogenesis. Moreover, evidence suggests that these pesticides disrupt embryogenesis and development, have long-term impacts on the structural and functional integrity of the nervous system, and may increase the risk of neurodegenerative diseases (33, 34). Organochlorine pesticides, which are commonly used in agriculture, can accumulate in the maternal body and be transmitted to the fetus through the placental barrier, potentially leading to birth defects (35).

This study identified a correlation between maternal pesticide exposure during the periconceptional period and cardiovascular system defects, consistent with prior research. A birth cohort study conducted in North Carolina found a significant association between maternal exposure to pendimethalin and an increased risk of atrial septal defect in offspring (OR = 2.09, 95% CI = 1.63–2.68), as well as an elevated risk of patent ductus arteriosus (OR = 1.64, 95% CI = 1.29–2.08) (36). Similarly, results from a nested case–control study suggested a trend toward a higher risk of septal defects (ORs ranged from 1.80 to 2.36) with greater serum neonicotinoid pesticides (NEOs), especially nitro-containing NEOs represented by imidacloprid (IMI) (37). Most animal studies have used nitrofen to induce congenital heart malformations in mouse embryos (38, 39), with the primary mechanism involving the impact of toxic substances on the proliferation or apoptosis of cells involved in heart development. Due to poor perceptions of biological control and lack of capacity, many low- and middle-income countries are reluctant to pursue biological control, increasing a dependency on pesticides, and increases the risk of pesticide exposure for mothers during pregnancy (40). In addition, since biopesticides can promote the increase of crop production without compromising human health, they have already received widespread attention (41). However, there are very few studies on the relationship between exposure to new pesticides during pregnancy and cardiovascular system defects, further research is needed to confirm its safety to mothers and children.

This research represented a large-scale, population-based cross-sectional survey conducted in Northwest China, focusing on the correlation between maternal pesticide exposure during the periconceptional period and the incidence of birth defects. However, several limitations should be acknowledged. Firstly, pesticide exposure was assessed retrospectively through self-reporting, which might introduce recall bias due to the time elapsed between pregnancy and the survey. To mitigate this bias, we established a rigorous investigative protocol and selected clear indices pertinent to pesticide exposure, thereby enhancing participants’ ability to accurately recall long-term exposure histories. For example, the indices encompassed commonly used local pesticides and their corresponding application seasons. Secondly, the low incidence of birth defects and the limited number of cases of malformations outside the cardiovascular system prevented an analysis of other types of birth defects. Thirdly, the study did not differentiate between specific types of pesticide exposures, limiting our ability to perform sensitivity analyses. Finally, despite adjusting for potential confounders using multivariable regression, unmeasured or latent confounders, such as pregestational diabetes mellitus, genetics, environmental toxins and other environmental risk factors, might still influence the results.

## 5 Conclusion

Our findings demonstrated that maternal exposure to pesticides during the periconceptional period was associated with an increased risk of birth defects, particularly those affecting the cardiovascular system in offspring. This association is particularly pronounced among individuals residing in rural areas and those undergoing subsequent pregnancies. Therefore, in order to prevent birth defects in infants, it may be beneficial to pesticide exposure from 3 months before conception through the first trimester of pregnancy. These findings carry significant policy implications for maternal health interventions in China.

## Data Availability

The original contributions presented in the study are included in the article/Supplementary material, further inquiries can be directed to the corresponding author.
