# Supplementary material for: Maternal pesticide exposure and risk of birth defects: a population-based cross-sectional study in China
Source: Front Public Health. 2024 Dec 6;12:1489365. doi: 10.3389/fpubh.2024.1489365 (PMC11659231; doi:10.3389/fpubh.2024.1489365)
Supplement: Supplementary file 1 [file Table_1.docx]

**Supplementary Table S1** Baseline characteristics of participants

| Variables | Total | Pesticide exposure group | Non-pesticide exposure group | t/χ^2^/Z | P value |
| --- | --- | --- | --- | --- | --- |
| Ethnic, n (%) |  |  |  |  |  |
| Han | 29017(99.36) | 338(99.71) | 28679(99.36) | 0.211 | 0.646 ^a^ |
| Other | 187(0.64) | 1(0.29) | 186(0.64) |  |  |
| Type of pregnancy, n (%) |  |  |  |  |  |
| Singleton | 28852(98.79) | 334(98.53) | 28518(98.80) | 0.209 | 0.647 |
| Multiple | 352(1.21) | 5(1.47) | 347(1.20) |  |  |
| Family history of birth defects, n (%) |  |  |  |  |  |
| Yes | 136(0.47) | 0(0.00) | 136(0.47) | 0.749 | 0.387 ^b^ |
| No | 29068(99.53) | 339(100.00) | 28729(99.53) |  |  |

^a^ Fisher exact test; ^b^ Continuity corrected χ^2^ test.

**Supplementary Table S2** Association between pesticide exposure and birth defects in subgroups

|  | Subgroups | Model 1 |  | Model 2 ^a^ |  | P for interaction |
| --- | --- | --- | --- | --- | --- | --- |
|  |  | Crude OR (95%CI), P value |  | Adjusted OR (95%CI), P value |  |  |
| Maternal age | <30 years | 3.78 (2.26, 6.33), <0.001 |  | 2.14 (1.20, 3.81), 0.001 |  | 0.830 |
|  | ≥30 years | 4.18 (2.35, 7.43), <0.001 |  | 2.65 (1.51, 4.65), <0.001 |  |  |
| Education | High middle school and above | 5.63 (2.01, 15.77), 0.001 |  | 2.99 (1.05, 8.56), 0.041 |  | 0.490 |
|  | Middle school and below | 3.70 (2.64, 5.20), <0.001 |  | 2.30 (1.64, 3.21), <0.001 |  |  |
| Residence | Rural | 3.57 (2.67, 4.75), <0.001 |  | 2.37 (1.83, 3.07), <0.001 |  | 0.332 |
|  | Urban | 6.94 (1.87, 25.84), 0.004 |  | 3.19 (0.87, 11.67), 0.080 |  |  |
| First pregnancy | Yes | 2.90 (0.95, 8.84), 0.062 |  | 1.64 (0.56, 4.80), 0.363 |  | 0.634 |
|  | No | 3.98 (2.79, 5.68), <0.001 |  | 2.57 (1.81, 3.64), <0.001 |  |  |

^a^ Model 2 adjusted baseline demographic characteristics, fertility status, nutritional factor, and environmental factors.
